# Rethinking Empowerment in Healthcare: A Dynamic Continuum

**DOI:** 10.1111/nin.70167

**Published:** 2026-09-19

**Authors:** Cathy J. Beresford

**Affiliations:** ^1^ Department of Nursing University of Sunderland in London London UK

**Keywords:** carers, co‐production, disempowerment, empowerment, healthcare, healthcare professionals, liver disease, patients

## Abstract

Empowerment is a widely used term in healthcare literature, policy and practice, but its meaning is often inconsistent and increasingly framed as an individual outcome linked to self‐management, responsibility and compliance. However, such framings risk oversimplifying empowerment and reinforcing neoliberal and paternalistic assumptions that place responsibility on individuals while overlooking wider relational, systemic and contextual influences. Drawing on findings from constructivist grounded theory research with people affected by advanced liver disease, carers and healthcare professionals, I propose that empowerment is not a fixed state or something that can be delivered by professionals, but rather a dynamic, relational process that exists on a continuum and frequently co‐exists with disempowerment. Rather than being an outcome to be achieved, empowerment is enacted through everyday actions, relationships, resistance and navigation of constraint across patients, carers and professionals. The paper proposes a reconceptualisation of empowerment as relational, contextual and co‐produced, with implications for moving away from notions of empowerment as professional intervention or individual achievement.

## 1 Introduction: Why Empowerment Needs Rethinking

The term ‘empowerment’ has been widely used in health and social care since the late 20th century after it was first introduced in relation to civil rights and political movements (Rappaport 1981; Freire 2018; Zimmerman 1995). Despite its broad use, the meaning of empowerment in healthcare is not universally accepted, and the wider literature illustrates complexities, tensions and assumptions. Some scholars have described empowerment as a goal or a measurable outcome (McAllister et al. 2012; Pekonen et al. 2020), whereas others have emphasised it as a process (P. Beresford and Croft 1993; Freire 2018). Health policy has leant towards outcome models of empowerment, framing it as achievable through provision of information, tools and services (NHS England c2025; c2026). It has also been identified as both a process *and* an outcome (Cerezo et al. 2016; Zarinbal et al. 2025).

Frequently, healthcare professionals have been encouraged to find ways to *empower* their patients, as if empowerment can be bestowed from one person to another (Ibrahim et al. 2024). The term is often used to suggest that patients *should* be empowered to manage their health and lives, with the implication that if they are not empowered, they have failed in some way.

There is a risk of empowerment being oversimplified, with an emphasis on individual responsibility, rather than consideration of the wider systemic and relational factors that impact on people's lives, such as access to support, services and constraints, such as illness. Furthermore, this neoliberal framing of empowerment as an individual responsibility and measurable outcome aligns with a paternalistic approach to healthcare, where the practitioner knows best and facilitates empowerment for the ‘unempowered patient’. The reality may be quite different. Recent PhD research with people affected by advanced liver disease found that empowerment is a dynamic, relational process which exists on a continuum rather than as a fixed outcome (C. J. Beresford 2025). Empowerment frequently occurs in tension with, or *despite* health and social care systems and relates to patients, families, carers and professionals, who all experience and contribute to empowerment in different ways.

This paper is informed by a constructivist understanding of knowledge (Charmaz 2025), in which empowerment is understood as socially produced, relational and context‐dependent rather than a fixed attribute of individuals. The aim of this paper is to critically explore the concept of empowerment and offer a theoretically informed, empirically grounded understanding that reflects real‐world complexities in healthcare. The paper draws upon insights from qualitative research with individuals with advanced liver disease, carers and healthcare professionals, which is reported in more detail elsewhere (C. J. Beresford 2025). Consideration includes the conceptual roots of empowerment, problems with contemporary uses of the term, exploration of what empowerment is and is not, how it exists alongside disempowerment, an empirically informed conceptualisation of empowerment, and implications for nursing, health and social care practice.

I propose three conceptual shifts:

1. From empowerment as outcome to empowerment as dynamic process.
2. From empowerment as bestowed to empowerment as enacted.
3. From empowerment versus disempowerment to empowerment alongside disempowerment.

## 2 Historical and Conceptual Roots of Empowerment

### 2.1 Origins in Civil Rights and Feminist Movements

In the 1960s, empowerment came to prominence in the Black civil rights movement, where it was expressed through struggles for autonomy, self‐determination and collective action (Solomon and Solomon 1976; Adams 1996). The principles of empowerment, particularly unity strength, control, action and decision‐making *within* individuals and communities, were crucial to the resistance and resilience of Black communities fighting oppression (Hamilton 2011). Freire (2018) later conceptualised these ideas more explicitly, describing empowerment as the development of critical consciousness, whereby individuals and communities recognise and challenge oppressive structures.

In the 1970s, feminists adopted empowerment through collective movements to argue for women's rights, strive for equality and challenge patriarchal structures emphasising the importance of collective action and resistance to structural inequality rather than individual advancement (hooks 1984). Around this time, empowerment began to feature within disability and mental health movements, where people challenged paternalistic models of care and advocated for greater autonomy, self‐determination and recognition of lived experience (Chamberlin 1978; Rappaport 1981). Together, these movements positioned empowerment not solely as an individual capacity to adapt to existing systems, but as a collective process of challenging unequal power structures and transforming the social conditions that perpetuate oppression.

### 2.2 Transfer Into Health and Social Care Discourse

Gradually, empowerment was adopted by social work (Adams 1996) and by the 1990s, it had gained prominence within nursing and healthcare literature, for example Freire's ideas influenced approaches to diabetes education that sought to support people in managing their condition and living well with diabetes (Funnell et al. 1991). Researchers considered how patients and service users could be empowered to manage their health and lives (Gibson 1991; Rodwell 1996). Empowerment was seen as an alternative to compliance, with patients considered responsible for choices around their health, rather than simply the recipients of medical decisions (Aujoulat et al. 2007).

Reading the early papers, one can see that the intentions were beneficent; an attempt to move away from paternalistic and hierarchical practice, towards approaches where the role of patients and service users were recognised, valued and prioritised (Rappaport 1981; Entwistle and Watt 2006). However, ironically, the idea that practitioners could ‘empower’ the people they provided care and support to may have inadvertently reinforced the paternalistic attitude that practitioners know best, positioning individuals as unable to empower themselves and in need of this being conferred upon them (Entwistle et al. 2018).

It also contributed to the moralisation of health behaviours, for example managing a long‐term condition such as diabetes ‘well’ was seen as evidence of empowerment and framed as ‘good’ (Hernandez‐Tejada et al. 2012), while ‘poor control’ was viewed as ‘bad’ (Broom and Whittaker 2004). This reflects wider ideas of healthism and individual responsibility where individuals are judged on their ability to manage their health (Broom and Whittaker 2004; Hosseini Nodeh et al. 2024). Terms such as ‘good’ or ‘bad’ diabetes management are still frequently used in clinical practice, education and research and have been critiqued as judgemental and stigmatising (Banasiak et al. 2020; Litterbach et al. 2024).

### 2.3 Competing Definitions and Theoretical Tensions

The complexity of empowerment as a concept has been widely documented (Gibson 1991; Anderson and Funnell 2010; Agner and Braun 2018). One influential definition from the World Bank (2007) conceptualises empowerment as the process of ‘enhancing the capacity of an individual or group to make purposive choices and to transform those choices into desired actions and outcomes’ (p. 6). This definition highlights that empowerment involves both agency and the social conditions that shape people's ability to act, rather than simply an individual attribute. Rodwell (1996) describes empowerment as: ‘In a helping partnership it is a process of enabling people to choose to take control over and make decisions about their lives. It is also a process which values all involved’ (p. 309).

While well‐intended, this framing suggests that people require enablement and do not already make choices and decisions, with the implication that empowerment is bestowed upon them rather than enacted by individuals or communities. In contrast, Gibson (1991) argues that healthcare professionals cannot empower people because people empower themselves.

The role of power has also been contested. Traynor (2003) and Rodwell (1996) suggest that empowerment represents a shift in power dynamics between healthcare professionals and patients, and Gibson (1991) argues that powerlessness is a defining aspect of *disempowerment*. Conversely, Anderson and Funnell (2010) state that ‘empowerment has nothing to do with giving or taking power’ (p. 279). However, power structures remain a defining component of empowerment throughout much of the healthcare literature, and this also applies to patient and public involvement (PPI) in research (Schilling and Gerhardus 2024), where empowerment is shaped by the ability of patients and communities to influence and contribute to decision‐making. Therefore, power *is* a central aspect of empowerment.

### 2.4 Towards a Reconceptualisation of Empowerment

The literature demonstrates persistent and unresolved tensions in how empowerment is conceptualised and applied within healthcare, including whether it is a process or an outcome, whether it is enacted by individuals or bestowed by professionals, and whether it operates in opposition to or alongside disempowerment (Gibson 1991; Rodwell 1996; Anderson and Funnell 2010; Agner and Braun 2018). These tensions suggest that empowerment is neither stable nor singular, but instead a fluid and contested concept that resists simple definition. In response, I propose a conceptual rethinking of empowerment: first, as a dynamic process rather than a fixed outcome; second, as something enacted by individuals and collectives rather than bestowed by professionals, and third, as a phenomenon that exists alongside, and in interaction with, disempowerment rather than in opposition to it.

The following sections explore these shifts, illustrating how empowerment is more usefully understood as a dynamic, relational and context‐dependent process, which co‐exists with disempowerment.

### 2.5 Empowerment as Outcome vs. Empowerment as Process

Empowerment in health and social care is often framed as an outcome, or an achievable static goal, with some researchers introducing tools and measures for it to be evaluated as part of assessing service success (McAllister et al. 2012; Pekonen et al. 2020). In this framing, empowerment is closely linked to self‐management, with greater independence in maintaining health and life often taken as an indicator of higher levels of empowerment (Hernandez‐Tejada et al. 2012).

However, framing empowerment in this way is problematic. For instance, it ignores the barriers that individuals may face when self‐managing their health, such as access to nutritious food and opportunities for physical activity. Furthermore, people with illness or impairment may have constraints as to how independent they can be. Consequently, this approach to empowerment risks reinforcing ableist assumptions that equate independence with success and dependence with failure (Shakespeare 2006; Mannor and Needham 2024). When empowerment is positioned in this way, it may marginalise those whose capacity varies and focuses on individual responsibility rather than a holistic understanding of the factors that contribute to self‐management.

In contrast to approaches that treat empowerment as a measurable outcome or a static goal, I argue that empowerment is better understood as a process and a continuum. This aligns with Freire (2018) and P. Beresford and Croft (1993), who conceptualise it an ongoing process rather than a destination or a static goal, and Gibson (1991) who highlights that empowerment is difficult to operationalise and cannot be fully captured through a single measure.

### 2.6 When Empowerment Becomes Compliance in Disguise

A pervasive theme within parts of the healthcare empowerment literature is the idea that ‘empowered patients’ are autonomous, able to make their own decisions, effective in self‐care and work collaboratively with healthcare professionals (Anderson and Funnell 2010; Hernandez‐Tejada et al. 2012). This includes attending appointments, taking medication and managing their condition in ways that align with professional expectations. Such behaviours are also framed as beneficial to healthcare services, as the perceived ‘burden’ of disease is reduced, patients require less support and are considered less likely to become more unwell (Hernandez‐Tejada et al. 2012; Small et al. 2013; Vaughan and Lavery 2024).

This version of empowerment is more aligned with traditional notions of healthcare where what is really being proposed is that the ‘empowered patient’ is more compliant and aligned with the agenda of healthcare providers (Aujoulat et al. 2007; Anderson and Funnell 2010). In this version, a patient who does not take their medication is considered ‘non‐compliant’. The reality may be different: the person may be exercising empowerment because they may have weighed up the advantages and disadvantages of the medication and decided not to take it because it does not align with their preferences and wishes (Koenig 2011). Nonetheless, Traynor (2003) illustrates that in healthcare empowerment, such resistance is rarely viewed as empowerment.

However, empowerment is not synonymous with compliance or adherence, although the application of it in healthcare can perpetuate this ideology (Traynor 2003). Rather, it is about exercising autonomy and making choices, which may or may not be considered ‘wise’ from a professional perspective. For example, a person who refuses treatment that may clinically benefit them may still be acting in an empowered way as control, autonomy and decision‐making remains with them. Agner and Braun (2018) point out that non‐compliance is linked to empowerment because it can be understood as a form of self‐determination and control. Similarly, Koenig (2011) found that patient resistance can be a way of asserting agency in treatment decisions, and an important aspect of active participation. This aligns with the research findings behind this paper (C. J. Beresford 2025), where individuals exercised empowerment through actions such as making formal complaints and changing healthcare providers when dissatisfied with services.

### 2.7 Empowerment and Responsibility: Shifting Burden Onto Individuals

A particularly dominant contemporary framing of empowerment in healthcare is shifting responsibility onto individuals by focusing on self‐care and self‐management (Small et al. 2013; Vaughan and Lavery 2024). These are presented as desirable not only for the benefit of those utilising healthcare services but also as a means of reducing the perceived ‘burden’ on health services and shifting responsibility for health outcomes onto individuals. Through a district nursing lens on patient engagement, Vaughan and Lavery (2024) argue that ‘the current NHS burden is unsustainable and depends upon patients, carers and families taking responsibility for their future health’ (p. 477). Similarly, Hosseini Nodeh et al. (2024) argue that future governments need to ‘adopt approaches in which people are empowered to take responsibility for their health and actively play a role in choosing healthy lifestyles’ (p. 2).

However, this framing can be challenged in two ways. First, it assumes that patients, carers and families do not already take responsibility for managing health and illness, an assumption contradicted by qualitative evidence from people living with advanced liver disease (C. J. Beresford 2025). Second, it does not adequately account for the wider socioeconomic, systemic and relational factors which contribute to health, illness and people's capacity to self‐manage (Gibson 1991; Department of Health and Social Care 2018; Entwistle et al. 2018). These include inequalities in finances, housing, education and access to services, which place constraints on the extent to which individuals and communities can realistically be able to enact ‘responsibility’. This critique aligns with Agner and Braun (2018), who argue that dominant constructions of empowerment inadequately address structural and context influences by focusing on individual responsibility.

Anderson and Funnell (2010) highlight that healthcare professionals are socialised to take responsibility for patient care and outcomes, while also arguing that patients are responsible for the majority of day‐to‐day management. While this distinction is important, it risks reinforcing a binary understanding of responsibility that overlooks the broader systemic, political and relational conditions within which both patients and professionals operate. These tensions contribute to the construction of an idealised ‘empowered patient’ while obscuring the relational and structural realities of healthcare practice.

### 2.8 The Myth of the ‘Empowered Patient’

Reading the literature, it becomes apparent that despite the claim that empowerment cannot be bestowed from one to another (Gibson 1991), it is frequently written about in ways that frame empowerment as something that can be enacted upon patients. For example, in their discussion of empowerment‐based interventions in diabetes care and education, Anderson and Funnell (2010) emphasise that empowerment is not something that can be done to patients. However, their focus on professional‐led approaches positions empowerment as something that can be facilitated through clinical practice.

This reveals a tension at the centre of how empowerment is frequently framed in healthcare literature and understood in practice. The focus is on patients reaching an empowered state where they can manage their health, make decisions and take an active role in their care. It is presented as something that requires the input of healthcare professionals to help patients reach the status of empowerment, with limited recognition of the capacity of individuals and communities to enact empowerment for themselves and the challenges they may face in doing so (Fricker 2007).

## 3 An Empirically Informed Conceptualisation of Empowerment

The conceptualisation of empowerment presented in this paper was informed by previous research (C. J. Beresford 2025). While the empirical findings are reported elsewhere, this research led to a deeper consideration of how empowerment is conceptualised in healthcare. In particular, it highlighted how empowerment is often framed in ways that promote compliance and individual responsibility, while overlooking the wider determinants of health and the ways patients, families, communities and healthcare professionals already exercise empowerment in their everyday lives and roles. Furthermore, it is rarely acknowledged that empowerment co‐exists alongside disempowerment.

In their discussion about empowerment in the management of Type 2 diabetes, Anderson and Funnell (2010) describe patient empowerment as a ‘process designed to facilitate self‐directed behaviour change’ (p. 277), and a specific healthcare approach that is the antithesis of compliance that professionals can use with their patients. Much of what Anderson and Funnell describe makes sense in the context of how they frame empowerment, for example, the idea that empowerment‐based interventions can help patients to learn critically and make informed decisions. However, a crucial aspect is missing: that empowerment already exists within patients who frequently find ways to exercise it despite the actions of professionals and the structures of services. Although Anderson and Funnell (2010) state that empowerment is ‘not something one does to patients’ (p. 277), their framing still presents empowerment in terms of what professionals do, such as providing empowerment‐based education.

This tension is perhaps unsurprising—it is challenging for us as healthcare professionals to switch our thinking from what we do, to what our patients do. But perhaps we have got it the wrong way around and are coming at empowerment from the wrong angle. Rather than focusing on it from our perspective, it might be more constructive to focus on it from the perspective of those who are living with associated health conditions and social issues.

In her work on epistemic injustice, Fricker (2007) provides a useful lens through which empowerment in this context can be understood. Healthcare discussions of empowerment have largely focused on how healthcare professionals can empower patients. In doing so, they risk overlooking the experiential knowledge of patients and carers about the ways they already act in empowering ways in their everyday lives. Consequently, empowerment becomes understood as something facilitated by professionals, rather than recognised as a dynamic process that people are already enacting despite the challenges they face.

This was evident in the research behind this paper, both in the public involvement work and in interviews with people living with liver disease. People often focused on what they did themselves to manage their health and illness. While the role of healthcare professionals and access to services was important, what emerged more strongly was how people made decisions, exercised autonomy and control over what was happening to them. While information, communication, professional relationships and navigating healthcare services were profoundly influential to people's experiences of care, their own role in advocating, deciding and acting were powerful.

This perspective resonates with earlier social movements which included empowerment at their core, for example, Black civil rights. It would be patronising and incorrect to suggest that white people empowered Black people. Hamilton (2011) argues that Black people empowered themselves through agency, self‐organisation and political self‐determination, and explicitly rejected reliance on white benevolence. Similarly, in the feminist movement of the 1970s, men did not empower women. Feminists emphasised organisational autonomy and self‐reliance, articulating and pursuing their own demands for liberation rather than relying on sympathetic male allies (Owen 2013). That is not to suggest that white people or men may not have a role or a contribution, but they are not the source of empowerment. As argued by Freire (2018), people are their own agents of change.

I present a similar stance regarding the empowerment of patients, arguing that empowerment is not something that is done to people. It begins with individuals' own actions and control over their daily lives, within the context of systems and relationships. Furthermore, this perspective can be extended to how we consider the empowerment of healthcare professionals. It is not only the patient empowerment literature that frames empowerment as something that is done to people. In a literature review on empowerment of nursing students in clinical practice, Kennedy et al. (2015) found several papers that presented workforce empowerment as an organisational tool used for the organisation's benefit. They also point out that such approaches do not always address the actual experience of empowerment by employees.

The empirical research informing this paper similarly found that healthcare professionals took active steps to empower themselves, rather than being empowered by managers or organisations (C. J. Beresford 2025). These included leading and collaborating on quality improvement projects, advocating, professional networking, continuous professional development, participating in research and teamworking. This suggests that professional empowerment emerges through individual and collective action, rather than being conferred solely through organisational structures.

Informed by this analysis and through active engagement with people living and working in the field of liver disease throughout the PhD, empowerment can be conceptualised as encompassing control, informed decision‐making, choice, rights and active participation. It is a dynamic and relational process that involves being supported, educated and having access to resources while also requiring strength, capacity and autonomy. Empowerment is shaped through relationships and may involve advocacy, resistance and challenging existing structures. It operates at both individual and collective levels and is relevant to patients, carers and healthcare professionals alike.

Empowerment should not be reduced to compliance, passivity, alignment with healthcare professionals' beliefs or agendas, or an individual responsibility undertaken in isolation. Importantly, empowerment does not mean individuals can or should be expected to do everything independently. Instead, it reflects the ways in which people act, decide and respond within the context of their lives and relationships, including by coming together through peer support, professional networks and working collaboratively.

## 4 Implications for Nursing and Healthcare Practice

### 4.1 Moving Beyond ‘Empowering’ as a Professional Task

An implication of this paper and the understanding of empowerment that has been presented is that healthcare professionals might move beyond thinking of ways they can ‘empower’ their patients and service users. Instead, to understand that empowerment exists regardless of the circumstances, often alongside disempowerment, and that we can be allies, supporters and advocates for the people we provide care to, but we cannot empower them. Instead, it is useful to focus on the realities of what we can deliver (within the constraints of healthcare services) in terms of person‐centred care, and to remember that seemingly small actions can profoundly affect people's experiences and capacity for empowerment. For example, introducing ourselves, providing information empathetically, being open and honest can instil a sense of dignity and control for the individuals we work with (C. J. Beresford 2025).

### 4.2 Facilitating Conditions for Empowerment

The empirical research informing this paper highlighted that empowerment processes are facilitated by a range of relational, structural and individual factors. These include access to appropriate services, support and information; empathetic, open and honest communication; opportunities for peer support and professional networking; collaborative relationships between professionals, carers, families and patients, and the development of self‐belief and motivation.

Importantly, empowerment did not arise simply from the presence of these facilitating conditions. Disempowerment could also lead to empowerment. When people found themselves in disempowering situations, for example, due to a lack of information or access to services, they frequently found ways to overcome this despite significant challenges. That is not to suggest that disempowering situations are positive—they were frequently distressing and oppressive—but empowerment emerged despite this, through individual and collective action.

## 5 Empowerment and Disempowerment as Co‐Existing Processes

Empowerment exists alongside disempowerment. Challenges can lead to empowerment but can also be disempowering. For example, a lack of information provision from healthcare professionals can be disempowering for patients but can also spur them to seek information themselves. Disempowerment can trigger empowerment but can also lead to further disempowerment. For example, in the case of a patient who was not given accessible information about his prognosis, his stepdaughter took steps to seek information and secure a diagnosis for him. In her account, there were incidents of both disempowerment and empowerment, illustrating that empowerment frequently exists despite systems, rather than because of them.

Other scholars have argued that empowerment and disempowerment are not mutually exclusive and can co‐exist within the same structures of care (Bury 1982; Nettleton 2021). However, it can also be argued that empowerment is frequently produced through the interaction of empowering and disempowering situations, not simply existing alongside them. A powerful example is that of Robert (not his real name) who developed advanced decompensated liver disease after many years of excessive alcohol use. He was extremely unwell and close to death in hospital. Robert described his hospital admission as, in many ways, an empowering experience because of the quality of care he received and the support he experienced from healthcare professionals. However, he also described disempowering aspects of his care, particularly at discharge, when he was given little information about his condition and recovery needs, despite being diagnosed with hepatic encephalopathy. At this point he was still very unwell, could barely walk and required carers. Robert described this as a very difficult time in his life. As he gradually recovered, he explained that the combination of these experiences motivated him to seek information for himself and make changes to his life. He contacted the British Liver Trust (name now changed to Liver UK) and began attending peer support meetings. He described this step as transformative: he made new friends, stopped drinking alcohol, undertook public involvement work and his illness at the time of interview remained fairly stable. This account illustrates how empowerment can emerge through the interaction of both empowering and disempowering experiences within healthcare systems, rather than being produced by either in isolation.

## 6 Avoiding Blame, Burnout and Moral Distress

Understanding empowerment in this way can help to avoid the blame, burnout and moral distress that can be experienced by patients, carers, families and professionals. Various authors have explored these concepts in relation to all those groups (Abdoli et al. 2020; Jannati et al. 2020; Čartolovni et al. 2021; Ananzeh et al. 2026). For example, Salari et al. (2022) highlight that moral distress is a significant challenge for nurses due to conflicts between providing ethical care and the challenges preventing it. One of the strongest findings from the study underpinning *this* paper was the importance of small actions making a big difference in people's everyday lives and practice.

If empowerment is framed as an overall outcome that individuals can fully achieve, this puts pressure on patients to manage their health independently and risks positioning them as having failed if they are unable to reach the status as an ‘empowered patient’. In reality, each day involves small achievements, as well as losses. For example, in the study, an individual with advanced liver disease described situations that were disempowering to him, including being unable to remember information and events (due to illness), feeling exhausted, finding it difficult to cope with multiple appointments and frustration with healthcare services. However, equally he expressed empowerment through various actions including seeking peer support and information, advocating for himself, asking questions and making formal complaints when he was dissatisfied with care.

Healthcare professionals in the study described the immense pressures they faced due to their workload, staff shortages, service limitations and wider factors. They had to find ways to cope with the challenges, which could be incredibly disempowering—for example, the knowledge that some patients received inadequate palliative and end‐of‐life care. However, professionals themselves found ways to exercise empowerment. For example, undertaking quality improvement projects, continuous professional development, collaborative working and networking, and seeking peer support.

This raises an important question: do such actions mean that a professional is ‘empowered’, or does it mean they are doing their best to cope with the everyday realities of practice? The stance on empowerment presented in this paper is that empowerment and disempowerment exist concurrently on a continuum, rather than as fixed or opposing states. Supporting patients, carers, families and professionals to identify and focus on what they can do and what they have control over, may help to avoid the blame, burnout and moral distress that are commonly experienced in health and social care.

## 7 Conclusion: Towards More Honest and Relational Uses of Empowerment

The reconceptualisation of empowerment presented in this paper has practical implications for us as healthcare professionals. The first is that we move away from the idea that we can empower our patients—empowerment is not something that can be bestowed from one person to another; this way of thinking is paternalistic and implies that professionals know best. Instead, we should recognise, support and avoid constraining the empowerment that patients, carers and healthcare professionals themselves *already* exercise. This requires attention to our everyday practices and interactions through which care is experienced, including how we communicate, share information, recognise expertise and work collaboratively with individuals, families and carers within the constraints of their, and our, circumstances.

A further implication is to avoid proposing that individuals *should* be empowered and that they have failed if they are not empowered. It is not an individual responsibility, but a pervasive force which exists in the most challenging of circumstances and is often underestimated. Furthermore, it is appropriate to question whether empowerment is a measurable outcome or a fixed goal—it is a collaborative, relational process affected by wider factors. It occurs on an individual, relational and systemics level and exists on a continuum. Empowerment can be supported (but not bestowed) by professionals, access to services, co‐ordinated care and collaborative processes.

One might question why it is important to think about these issues. In some ways the term and ideas may seem abstract. However, in this paper I have illustrated that closer examination is important because it helps us to understand how we can support our patients, their families as well as each other. It is about avoiding blame for a lack of empowerment on individuals and instead working together to improve systems, processes and access to services. It is about sharing responsibility, not an individual burden, which can lead to burnout, guilt, shame, blame, the breakdown of professional relationships and loss of trust. Rather, it helps us all as patients, families, carers and professionals to focus on what we *can* control, what we *can* contribute and accept what is out of our control. In practice, this means recognising that seemingly small actions matter. While the big issues are important, for example, a lack of access to services, everyday interactions can profoundly impact empowerment. For example, the way we communicate with individuals and families can instil a sense of control, dignity and autonomy. It also means recognising the value of collective action. Whether through patient peer support or healthcare professional networks, empowerment is strengthened by working collectively. The support, information and sharing of good practice make a big difference. In summary, empowerment is not a professional intervention, nor an achieved status, but a fluctuating relational force shaped by context, constraint and collective action.

## Ethics Statement

Before recruitment, ethical approval to undertake the PhD study was obtained from the Proportionate Review Sub‐committee of the West of Scotland Research Ethics Committee (REC) 4 (IRAS project ID: 331914, REC reference: 23/WS/0150). Approval was also received from Bournemouth University (Ethics ID: 52129). All individuals who participated in the research did so with informed consent.

## Conflicts of Interest

The author declares no conflicts of interest.

## Disclosing the Use of Artificial Intelligence

In preparing and revising this manuscript, ChatGPT (OpenAI) was accessed between February 2026 and August 2026 to support clarity of expression, organisation of content and critical reflection on the presentation of ideas. The conceptual arguments, interpretations, analyses and recommendations presented in this manuscript are the author's own and arise from a co‐produced research project involving patients, carers, healthcare professionals and PhD supervisors. All content was reviewed, revised and verified by the author, who takes full responsibility for the final manuscript.

## Data Availability

The data that support the findings of this study are openly available in the Bournemouth Online Research Data Repository at https://doi.org/10.18746/bmth.data.00000519.
